# Detection of event-related potentials in individual subjects using support vector machines

**DOI:** 10.1007/s40708-014-0006-7

**Published:** 2014-11-25

**Authors:** Hossein Parvar, Lauren Sculthorpe-Petley, Jason Satel, Rober Boshra, Ryan C. N. D’Arcy, Thomas P. Trappenberg

**Affiliations:** 1 Faculty of Computer Science, Dalhousie University, 6050 University Avenue, P.O. Box 1500, Halifax, NS B3H 4R2 Canada; 2 Biomedical Translational Imaging Centre (BIOTIC), IWK Health Centre, Halifax, NS Canada; 3 School of Psychology, Faculty of Science, University of Nottingham Malaysia Campus, Semenyih, Selangor Malaysia; 4 Applied Sciences, Simon Fraser University, Surrey, BC Canada

**Keywords:** Support vector machine, Event-related potentials, Mismatch negativity, Electroencephalography, Neuroscience, Diagnostic

## Abstract

Event-related potentials (ERPs) are tiny electrical brain responses in the human electroencephalogram that are typically not detectable until they are isolated by a process of signal averaging. Owing to the extremely smallsize of ERP components (ranging from less than 1 μV to tens of μV), compared to background brain rhythms, statistical analyses of ERPs are predominantly carried out in groups of subjects. This limitation is a barrier to the translation of ERP-based neuroscience to applications such as medical diagnostics. We show here that support vector machines (SVMs) are a useful method to detect ERP components in individual subjects with a small set of electrodes and a small number of trials for a mismatch negativity (MMN) ERP component. Such a reduced experiment setup is important for clinical applications. One hundred healthy individuals were presented with an auditory pattern containing pattern-violating deviants to evoke the MMN. Two-class SVMs were then trained to classify averaged ERP waveforms in response to the standard tone (tones that match the pattern) and deviant tone stimuli (tones that violate the pattern). The influence of kernel type, number of epochs, electrode selection, and temporal window size in the averaged waveform were explored. When using all electrodes, averages of all available epochs, and a temporal window from 0 to 900-ms post-stimulus, a linear SVM achieved 94.5 % accuracy. Further analyses using SVMs trained with narrower, sliding temporal windows confirmed the sensitivity of the SVM to data in the latency range associated with the MMN.

## Introduction

Event-related potential (ERP) components are specific deflections in the averaged waveform, which are considered to be tied to discrete stages of neural processing. They are extracted from *trials* or *epochs* of electroencephalogram (EEG) data that are temporally locked to a repeated event, either a stimulus or a behavioural response, which are segmented from the continuous EEG recording and then averaged together. This averaging process reduces the amplitude of brain activity that is unrelated to the event, thus presumed to be random with respect to it in time, and retains brain responses that are temporally related to the event (see [1] for a full description of ERP derivation). The most commonly used definition of an ERP component was outlined by Näätänen and Picton in 1987 [2]. Their definition emphasizes that an ERP component is described not only by the brain regions that contribute to its production, but also by the experimental parameters which must be satisfied in order to observe the response. ERPs can be evoked by stimulation in any sensory modality and can also occur in response to motoric and cognitive events.

In the auditory domain, ERPs can be used to index processing all the way from early sensory processing in the brainstem to cortical language processing. The auditory brainstem response (ABR) has a long history of use in both neurology and audiology [3]. Cortical ERPs have yet to become a routine part of clinical practice, even in audiology (reviewed in [4]) and cognitive assessment of higher functions like language [5–7] where their potential utility is very clear.

In large part, the failure of cortical ERPs to be translated into clinical practice is due to the requirements of the signal-averaging process. Many repetitions of the evoking stimulus or event must be presented in order to isolate ERPs, which can lead to long testing times. Another important consideration for the application of ERPs for both clinical and research purposes is the need to statistically verify the presence of the response. Given the low signal-to-noise ratio of these responses versus background EEG, the typical approach in ERP research is to perform univariate statistics across groups, or across experimental conditions, using amplitudes that are measured in the averaged waveform [8].

Many groups have proposed solutions to verify the presence of ERPs in individual subjects, and even single epochs, by applying techniques such as wavelet analysis [9–11], independent component analysis [12], integrated waveforms [13], and nonparametric analyses [14]. In some very specific clinical situations, the approach of repeating stimulation until a statistical threshold is reached [15], or using basic t-scores to evaluate the presence or the absence of a particular component [16, 17], has been examined.

In particular, the field of Brain–Machine Interfaces (BMIs) has yielded much progress in the single-subject detection of responses like ERPs. This area seeks to use brain imaging data, mostly noninvasive technology, such as EEG, to identify brain waves in continuous EEG recordings that can be interpreted as commands by a computer [18]. The progress that has been seen in this area is mainly due to advances in machine learning techniques. While results in BMI research show that signals can be extracted from single trials, here we investigate specifically the use of machine learning techniques for the detection of ERPs in individual subjects for the purpose of clinical evaluations. Support vector machines (SVMs) are evaluated for their applicability as part of the protocol known as the Halifax Consciousness Scanner [19]. Our approach is hence mediating between a classical clinical approach and methods of BMIs.

The SVMs are a form of supervised statistical machine learning (for a primer, see [20]) and were first described by Vapnik and colleagues in 1992 [21]. When classified cases are used to train an SVM, it derives an optimal function based on the features that separate the two classes. Unlike other statistical classification methods, such as discriminant function analysis and linear regression, SVMs derive their decision function from only a subset of the data—the cases that are difficult to classify. These cases are referred to as support vectors. The goal of an SVM is to maximize the distance between the decision function’s boundary and the support vectors. They can operate in either a linear or a nonlinear fashion and are notable among machine learning techniques in view of their ability to produce generalizable models from small datasets [22].

The SVMs have demonstrated very high accuracy for discriminating between experimental conditions [23], and moderately high accuracy for the prediction of clinical group membership [8] based on ERP features. The present work arose from the development of a novel method for evaluating brain function using ERPs, known as the Halifax Consciousness Scanner (HCS; described in [19]). The initial intent of the HCS is for applications in brain-injured populations. This context presents very different challengesfrom BMIs because patients may be unable to comply with task instructions [24]. It therefore uses stimulation parameters that do not require effortful task completion. This approach is also beneficial for clinical populations that may seek to influence their test results to conceal, augment, or even fake an injury [25]. Yet another important feature that distinguishes clinical applications from typical SVM applications in BMIs is the need for rapid ERP detection, without any kind of training period for the classifier. For this reason, we apply SVM training across subjects, rather than in the within-subject fashion that is typical for BMIs.

To evaluate the ability of an SVM to verify ERPs in individual subjects, the present study uses an ERP known as the mismatch negativity (MMN). First discovered by Näätänen and colleagues in 1978, the auditory MMN is an ERP that indexes the detection of change in a sequence of sounds [26]. The MMN is linked to a level of auditory memory that is believed to form the basis of conscious perception [27], and is an important part of the HCS method [19], particularly due to its well-established utility for coma monitoring [15, 28–32]. Owing to its small size, the MMN presents a challenge for detection at the individual subject level.

The MMN is commonly evoked using an experimental design known as an oddball paradigm. Although it is well understood that the oddball paradigm frequently causes an undesirable overlap of other ERPs with the MMN [33], it provides a simple framework for understanding how the MMN is obtained. In the oddball paradigm, a sequence of identical “standard” stimuli is interrupted at random and unpredictable times by “deviant” stimuli that possess some changed features, such as tonal frequency, intensity, or duration. The MMN is observed as an enhancement in negative voltage that occurs approximately 100–250 ms from deviant stimulus onset. It rests atop other ERP components that are associated with sensory processing, such as the “obligatory” P1–N1–P2 complex, and is isolated from these responses through a process of subtraction in which the averaged waveform to the standard stimuli is subtracted point-by-point from the averaged waveform to the deviant stimuli. This produces the so-called “difference wave,” in which the MMN appears as a negative deflection in the 100–250 ms range. Although the amplitude of the MMN can be enhanced by experimental parameters such as reducing the silent period between stimuli (the interstimulus interval, or ISI), or increasing the difference between the standard and deviant tones [34], it is often quite small in amplitude, being around 1 μV.

The MMN is not restricted to simple oddball deviance and can also be evoked by more complex violations of concrete or abstract rules [35]. Under these circumstances, a pure MMN should be obtained, which is free of overlap from other ERP components. In the present study, the MMN is evoked in response to violations of a simple pattern of two alternating tones. Two types of SVM analyses are performed: one to achieve high classification accuracy (SVM_acc_), and the other to help one clarify whether the waveform features that are associated with the MMN are indeed used by the SVM (SVM_time_). The SVM_acc_ analysis explores a variety of factors which could impact classifier accuracy: number of epochs entered into the averaged waveforms, scalp electrode selection, kernel type, and temporal window selection.

It is generally accepted that, as the number of epochs entered into the ERP waveform increases, the SNR of the ERP improves versus background EEG [1]. For the MMN, the number of epochs that is generally recommended for averaging is at least 150 [36], although robust MMNs have been observed with approximately 60 [34]. For this reason, the first SVM analysis explores the influence of number of epochs entered into the average on the accuracy of the classifier.

The typical scalp distribution for the MMN is maximal at frontal sites, and inverted at sites below the Sylvian fissure when a nose reference is used. This distribution has been attributed to bilateral, vertically oriented cortical generators on the supratemporal plane, as well as a frontal source [37]. Thus, a classifier that is limited to frontal sites might achieve the highest performance. However, for clinical populations such as patients with acquired brain injuries, atypical scalp distributions are expected [38]. For this reason, the value of using scalp electrode sites separately, or combined, is evaluated.

The so-called kernel trick permits SVMs to operate in a nonlinear fashion by projecting low-dimensional data into higher-dimensional space [20]. This may provide a better fit to complex datasets, but this improved accuracy may come at the cost of overfitting—a lack of generalizability of the results to new datasets [20]. For this reason, a linear classifier may be preferable, but the values of different kernel types are examined to determine whether significant improvements in performance can be achieved over the linear solution.

The MMN is expected to occur approximately 100–250 ms from stimulus onset [36]; however, other information in the ERP waveforms may be useful for classification. The portion of the waveform that is entered into the feature vectors is therefore varied, to determine what temporal window produces the maximum performance. A difficulty in the interpretation of SVMs, however, is that they derive their rules using a complex combination of factors. Any waveform features that are within the selected temporal window, be they part of the MMN or not, can contribute to the decision function. Thus, the SVM_time_ analysis is performed, not to achieve high accuracy, but to provide finer detail regarding which temporal regions of the waveforms contain detectable information for classification.

## Method

### Subjects

One hundred healthy adult volunteers (57 females, aged 19–73, mean = 32.5 years) with no history of neurological problems, hearing problems, or psychoactive medications participated in the study. The study was carried out according to the Canadian Tri-Council guidelines (Health, Natural, and Social Sciences) on ethical conduct for research involving humans.

### The stimulus sequence

**Fig. 1:**
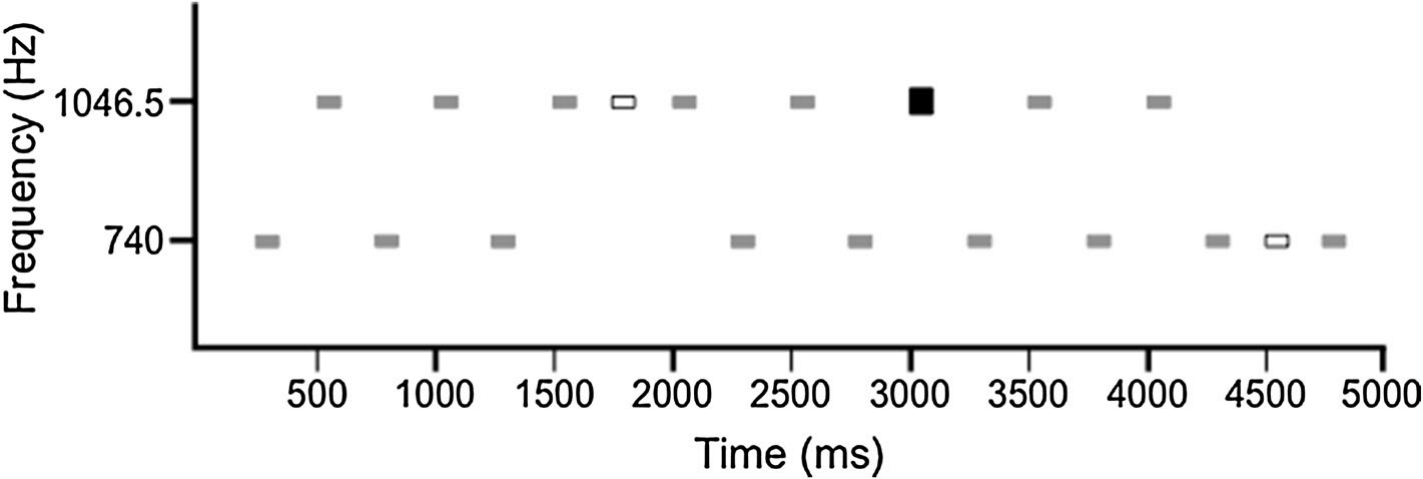
An illustration of a segment of the 2.5-min tone sequence. *Gray boxes* are “standards” that conform to the pattern. Type A and type B tones have fundamental frequencies of 740 and 1,046.5 Hz, respectively. The *open box* is a repetition deviant stimulus. The thicker, *black box*, is an intensity deviant. Both repetition deviants and intensity deviants can have either a type A or type B fundamental frequency

A segment of the tone sequence is illustrated in Fig. 1. This sequence was developed to evoke several ERPs for clinical applications such as brain injury [19]. It is composed of two spectrally rich tones (A and B) that consist of fundamental frequencies (740 and 1,046.5 Hz, respectively) with the 1st and the 2nd harmonics presented at exponentially decreasing levels (e^−1^ and e^−2^, respectively). These tones had durations of 100 ms, including a 5-ms total rise/fall time, and were delivered with an onset-to-onset interstimulus interval of 265 ms. The “standard” tones of the sequence were presented in an alternating pattern (ABABAB…) with an intensity of 75 dB SPL. Two types of deviant stimuli occurred infrequently within this standard pattern: repetition deviants (repetitions of either the A or the B tone, e.g., ABABBBA) and intensity deviants, both of which occurred with type A and type B fundamental frequencies. The sequential probabilities of repetition deviant and intensity deviant stimuli were 0.11 and 0.063, respectively. Tones were delivered in a fixed order in which consecutive deviant stimuli (regardless of type) were separated by at least two standard tones. In total, the tone sequence consisted of 601 stimuli, consisting of 496 standard tones, 67 pattern deviants (e.g., ABAB**B**BA), and 38 intensity deviants, and lasted for 2.5 min.

### Data acquisition and analysis

During sound presentation and EEG recording, subjects sat in a dimly lit room and watched a silent video with no subtitles. Subjects were asked to ignore all auditory stimulation and focus on the video. The EEG was recorded using a portable acquisition system consisting of a GmobiLab + 8 channel amplifier (g.tec Medical Engineering, GmbH), a custom-built triggering card, and a netbook. This configuration produced a small deflection in the EEG trace that was used to confirm the timing of stimulus delivery. Tin electrodes were placed at three scalp sites, including a Central site (at Cz), a Frontal site 5.8 cm anterior to Cz, and a Parietal site 5.5 cm posterior to Cz. Assuming a distance of 35 cm between the nasion and inion, these Frontal and Parietal sites would lie approximately halfway between Fz and FCz, and Pz and CPz, respectively, according to the extended 10–20 system. Additional electrodes were placed at the right mastoid and on the tip of the nose. This configuration of a portable EEG system with a reduced electrode montage was optimized for point-of-care clinical applications [19]. The vertical and horizontal EOG were recorded from two electrodes positioned on the supra-orbital ridge and outer canthus of the left eye, respectively. All sites were referenced to the left earlobe, and all impedances were below 5 kΩ. The EEG and EOG signals were sampled at a rate of 256 Hz, with a bandpass of 0.1–100 Hz, and stored for offline analyses.

Offline, these data were low-pass filtered at 20 Hz and notch filtered at 60 Hz. Signals were re-referenced to the nose, and eye movement and blink artifacts were corrected using a regression method [39]. The continuous EEG was then segmented into discrete 1,000-ms epochs, including a 100-ms prestimulus period, and any epochs containing values exceeding ±75 μV were considered to contain artifacts and were rejected from further analysis. For the ERP analysis, all epochs were averaged, and baseline corrected using the 100-ms prestimulus baseline. For greater detail regarding these standard ERP-processing steps, the interested reader is referred to [1]. In accordance with standard procedures for MMN analysis (e.g., [40]), the first four standard tones of the sequence, and the first 2–3 standards following every deviant were omitted from averaging. One participant’s mastoid electrode became disconnected during the session; therefore the mastoid data from this subject were excluded from the grand average.

Averaging for the SVM analysis that explored the impact of the number of epochs on accuracy was performed using 2–40 epochs for each stimulus type. An upper limit of 40 epochs was selected as this was the lowest number of deviant epochs available per subject in the 100-subject dataset. The data used for the SVM analysis did not receive baseline correction.

Similar to other research using two-tone alternating patterns [34, 40], standard and repetition deviant stimuli were averaged across tonal frequency stimulus types (e.g., all standard tones, regardless of whether they were low (A) or high (B) tones, were averaged together). The mean number of epochs accepted for averaging for each of these stimulus types was 214 for standard tone epochs and 66 for repetition deviant epochs. Data from the intensity deviant epochs, which evoke much larger ERPs and therefore do not test the sensitivity of the SVM approach, are not presented here.

### ERP scoring

The mean amplitude of the MMN was measured in a ±10-ms period surrounding its peak in each subject’s repetition deviant minus standard difference wave. All amplitudes were measured versus baseline.

### Group level statistical analysis

The MMN was tested for significance at the group level using a repeated-measures ANOVA with the factors electrode site (Frontal, Central, and Parietal) and stimulus type (repetition deviant and standard). Greenhouse–Geisser corrections were applied for all violations of the assumption of sphericity.

### SVM analysis for wave classification: SVM_acc_

#### Pre-processing

**Fig. 2:**
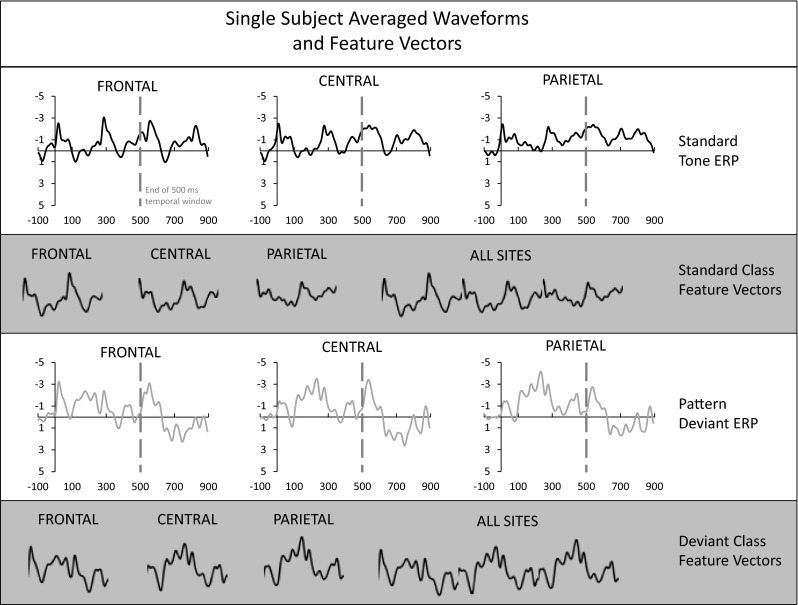
An illustration of averaged waveforms and feature vectors for a single subject. The upper panel shows standard tone averaged waveforms at the three scalp sites (Frontal, Central, and Parietal) using all available epochs. All amplitudes are in microvolts and all time values are in milliseconds (horizontal axes). The end of a 500-ms temporal window is delimited by a gray dashed line. The middle upper panel demonstrates how the data from that temporal window are used to create Frontal, Central, Parietal, and all scalp sites feature vectors for the standard ERP class. The lower middle panel shows pattern deviant averaged waveforms. Again, a 500-ms temporal window is delimited, and the composition of the Frontal, Central, Parietal, and all scalp sites feature vectors are shown, now for the deviant ERP class, in the bottom panel

Feature vectors for SVM training consisted of two types of waveforms: separate ERPs for each scalp site, and a vector in which the data from the three scalp sites were appended in a Frontal to Parietal order (referred to as all sites). For the exploration of temporal window size, data from a prescribed temporal window within the averaged ERP waveform were used. These windows always began at stimulus onset (*t* = 0 ms), and varied from 20 to 900 ms in width in 20-ms steps. The features in each vector were EEG samples (sampled at 256 Hz), therefore the number of features in each vector varied as a function of the size of the temporal window, and the number of electrode sites included in the vector. Illustrations of the types of feature vectors that were entered into the SVM analysis are provided in Fig. 2.

#### SVM training

Supervised learning was performed by 10-fold cross-validation over 100 datasets (i.e., 100 subjects) to predict the fit of the model to a hypothetical validation set. In explicit terms, this means that in each fold, the SVM was trained on 90 subjects, and tested on 10 *entirely different* subjects that were held out.

First, to explore the value of the number of epochs that are entered into the average using a simple model, first, linear SVMs were trained with waveforms containing data from all sites. Different numbers of epochs were entered into the average (i.e., one standard tone epoch and one deviant tone epoch, or averaged standard and deviant waveforms consisting of 2, 3,…,40 epochs). In separate analyses, these epochs were either randomly selected from amongst the available set of epochs, or selected in serial order. As described in Sect. 2.3, the maximum number of epochs entered into these averages was 40 due to the fact that this was the lower limit of available deviant epochs within the 100 subject dataset.

Following the determination that SVM accuracy increased as the number of epochs entered into the average increased, averaged waveforms consisting of all available epochs were used as feature vectors to determine the influence of kernel type, temporal window size, and electrode selection. A series of SVM analyses were performed that included three factors: kernel type (linear, radial basis function [RBF], polynomial-cubic, and polynomial-quadratic), window size (between 100 and 900 ms in 20-ms steps, beginning at stimulus onset), and all electrode sites. All default values were retained for the different SVM kernels (e.g., linear SVM soft margin box constraint *C* was left at 1, and radial basis function scaling factor *σ* = 1 was retained).

The final SVM_acc_ solution that provided the best performance was finally evaluated using a standard permutation analysis, as described in the introduction of [41]. The analysis was carried out with 1,000 permutations. The SVM implementation used for this study was the built-in SVM toolbox in Matlab 2012b (MathWorks Inc., 2012). Autoscaling was enabled, which normalizes all input data vectors to have unit standard deviation and centers them around their mean.

### SVM analysis to confirm MMN sensitivity: SVM_time_

#### Pre-processing

Averaged waveforms from all sites were used for the SVM_time_ analysis. Within this waveform, a 5 sample (approximately 20-ms) temporal window was defined, which slid by 5 sample steps across the entire 900 ms post-stimulus epoch.

#### SVM training

Linear kernel SVMs were trained to distinguish between pattern deviant and standard tone averaged waveforms (all available epochs) using data from each 20-ms sliding window, to a total of 46 windows/SVMs. As in the SVM_acc_ analysis, 10-fold cross validation was used across the 100 subjects. Again, the SVM implementation used for this study was the built-in SVM toolbox in Matlab 2012b (MathWorks Inc., 2012) with autoscaling enabled and the soft margin box constraint set to the default of 1.

## Results

### Group level ERP analysis

**Fig. 3:**
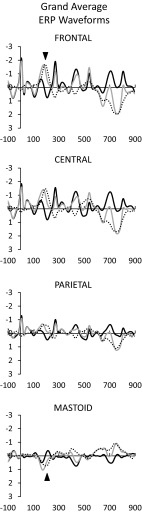
Grand average waveforms for standard tones (*black*), repetition deviants (*gray*), and the deviant minus standard difference wave (*dotted*). All amplitudes are in microvolts and all time values are in milliseconds. A sharp hardware-related deflection was observed at stimulus onsets (i.e., at 0 ms and every 265 ms thereafter). The MMN is indicated at the Frontal and Mastoid sites by an *arrow*. In the standard stimulus waveforms, obligatory auditory responses including P1, N1, and P2 were apparent. Because of the rapid rate of stimulus delivery, these took on the appearance of a steady state response

Grand average waveforms for standard and deviant tones, as well as the deviant minus standard difference waves, are shown in Fig. 3. In the repetition deviant waveform, N1 was followed by an additional negativity that was maximal at the Frontal site and reversed in polarity at the mastoid. This is the MMN, which is isolated in the deviant minus standard difference wave. The average peak latency of the MMN was 193.0 ms from stimulus onset. The ANOVA for the MMN demonstrated a significant effect of stimulus type [*F*(1,99) = 97.7, *p* < 0.001, *η*_partial_^2^ = 0.50] in which the mean amplitude at the latency of the MMN was more negative for repetition deviant than standard stimuli, thus, a significant MMN was evoked. There was also a significant effect of electrode site [*F*(2,198) = 21.2, *ε* = 0.63, adj. *p* < 0.001, *η*_partial_^2^ = 0.18], in which amplitudes were more negative at the Frontal site and the Central site than at the Parietal site. These main effects were qualified by an interaction [*F*(2,198) = 42.1, *ε* = 0.70, adj. *p* < 0.001, *η*_partial_^2^ = 0.30] in which the MMN amplitude in the repetition deviant waveforms was larger at the Frontal site than the Parietal site, but not the Central site. The mean amplitude of the MMN, measured at the Frontal site in the deviant minus standard difference wave, was −1.87 μV (s.d. = 1.50 μV).

### SVM_acc_ analysis

**Fig. 4:**
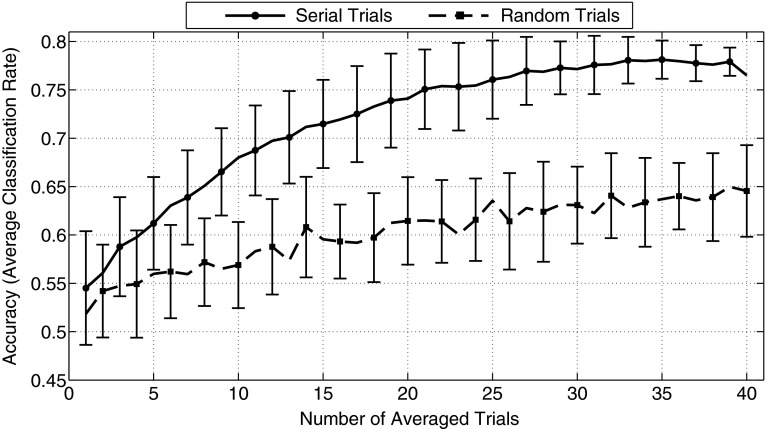
Accuracy of a linear SVM as a function of the number of standard and deviant tone epochs entered into the average (using all scalp sites). Accuracy increased as the number of epochs entered into the average was increased from 2 to 40, but increased much more steeply in the serial- than the random-selection condition, and reached a higher maximum when 40 epochs were used (78.13 %, SD = 0.02 % for serial selection, versus 65 %, SD = 0.043 % for random selection). *Error bars* indicate standard deviation

As can be seen in Fig. 4, the accuracy of the linear SVM increased as a function of the number of epochs entered into the average using both randomly and serially selected epochs. However, accuracy rose much more sharply as a function of number of epochs for the serially than randomly selected condition. In a similar vein, the maximum accuracy that was achieved for serially selected epochs (78.13 %, SD = 0.02 %) was substantially higher than that which was obtained using randomly selected epochs (65 %, SD = 0.43 %). Based on these results, further SVM analyses were performed on averages of all available epochs.

**Fig. 5:**
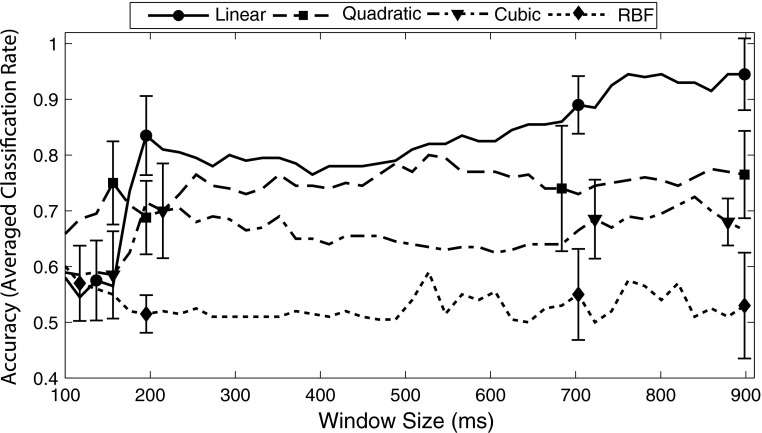
Effects of SVM kernel type and temporal window size for a feature vector that contains all three scalp electrodes (average of all available epochs). At window sizes below 180 ms, the quadratic kernel (*dashed line* *with* *square*) provided maximum accuracy, and was significantly better than other kernels. At a window size of 200 ms, however, the linear classifier (*solid line* *with* *circle*) rose above all other classifier types. Beginning at the 700-ms temporal window, the linear SVM provided significantly better performance than any other kernel type, and reached a maximum accuracy of 94.5 % (SD = 0.064 %) when the full post-stimulus epoch (900 ms) was used. For clarity, select *error bars* are displayed around 180, 200, 700, and 900-ms window sizes. *Error bars* indicate standard deviation

**Fig. 6:**
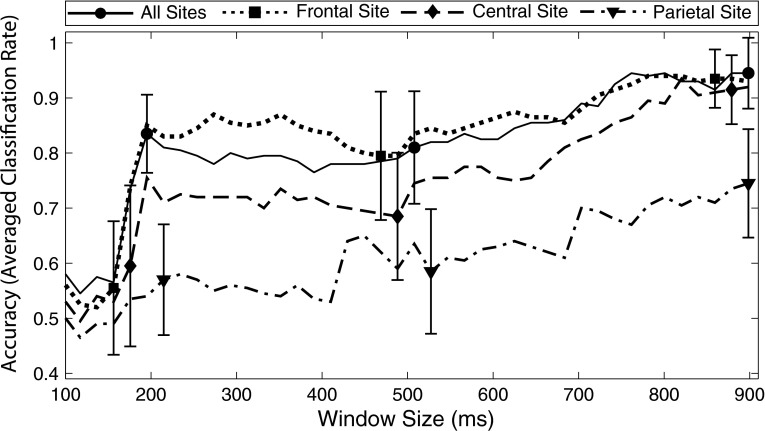
Effects of electrode selection and temporal window size on a linear SVM trained with feature vectors that contain all three scalp electrodes (average of all available epochs). As in Fig. 5, increasing the window width systematically improved classifier accuracy. At the largest window size, the Frontal site (*dotted line with diamond*), Central site (*dashed line with square*), and the feature vector containing data from all sites (*solid line with circle*) produced significantly better performance than the Parietal site (*dash-dotted line with triangle*). Nearly equal performance was obtained for the Frontal site, Central site, and all sites. For clarity, select *error bars* are displayed around 200, 500, and 900-ms window sizes. *Error bars* indicate standard deviation

The results of varying kernel type, window size, and electrode selection are presented in Figs. 5 and 6. Figure 5 demonstrates the effect of kernel type and window size for a feature vector that contains all scalp electrodes. At window sizes below 180 ms, the quadratic kernel provided maximum accuracy, and was significantly better than cubic or linear kernels. At a window size of 200 ms, however, the linearclassifier rose above all other classifier types, and performed significantly better than RBF or cubic classifiers. Unlike other kernel types, the accuracy of the linear SVM rose systematically as window size increased. Beginning with a 700-ms temporal window, the linear SVM provided significantly better performance than any other kernel type, and reached a maximum accuracy of 94.5 % (SD = 0.064 %) when the full post-stimulus epoch (900 ms) was used.

Figure 6 demonstrates the effect of electrode selection on a linear SVM, along with the effect of temporal window size. As in Fig. 5, it can be seen that classifier accuracy significantly improved when the temporal window reached at least 200 ms in width. Increasing the window width systematically improved classifier accuracy for SVMs trained at any site, but at the largest window size, the Frontal site, Central site, and the feature vector containing data from all sites produced significantly better performance than the Parietal site. Nearly equal performance was obtained for the Frontal site (93 %, SD = 0.048 %), Central site (92 %, SD = 0.063 %), and all sites (94.5 %, SD = 0.064 %).

**Fig. 7:**
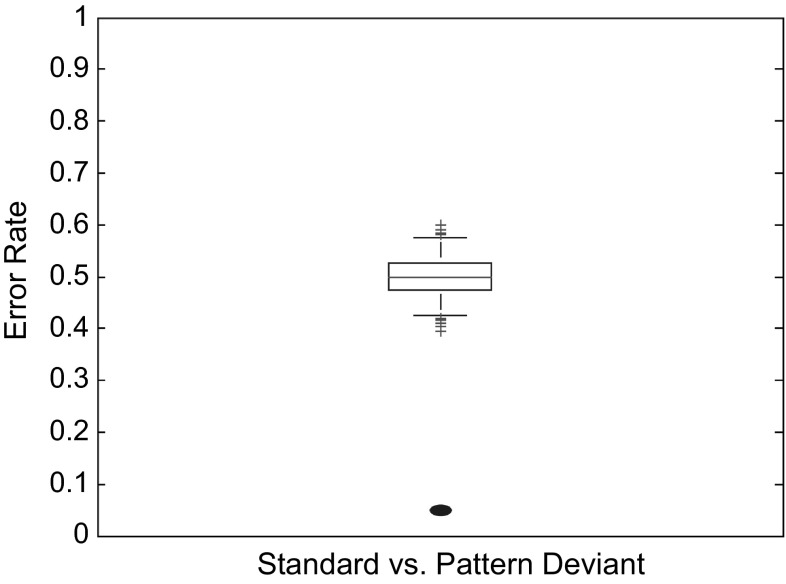
Box and whisker plot for the null distribution produced by the permutation test for the final SVM_acc_ solution (linear SVM, average of all available epochs, all sites, temporal window 0–900 ms). The center of the box is the median of the distribution. Box limits are the 25th and 75th percentiles, while the whiskers extend to the 2nd and 98th percentiles to delimit a two-tailed 4 % threshold for significance. Accuracies that are observed in the null distribution, which exceed these limits are indicated by *red cross marks*. The observed accuracy of the true SVM solution (mean error = 5.5 %) is indicated by a blue ellipse. (Color figure online)

The error of the final SVM_acc_ solution (linear SVM, temporal window 0–900 ms, all sites) was evaluated using a permutation analysis [41], the results of which are shown in Fig. 7. The *p* value for the accuracy of the true SVM solution was 0.001, and was outside the 96 % confidence interval of the accuracies that were observed with randomly assigned feature vectors.

### SVM_time_ analysis

**Fig. 8:**
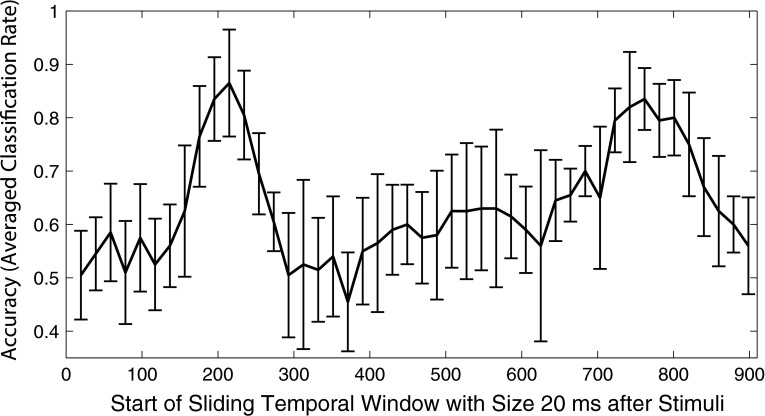
Accuracies of 46 linear SVMs trained using data in nonoverlapping 20-ms sliding temporal windows across the entire 900-ms post-stimulus period of the averaged waveform (all available epochs, all sites). The highest accuracies were achieved in the 160–260 and 720–820 ms periods (maximum 86.5 and 83.5 %, respectively)

Accuracies within each of the nonoverlapping temporal windows are depicted in Fig. 8, and ranged from a minimum of 45.5 % (SD = 0.09 %) to a maximum of 86.5 % (SD = 0.01 %). The highest accuracies were observed in the 160–260-ms and 720–820-ms periods.

## Discussion

The tone sequence evoked a moderate-sized, frontally maximal MMN component that inverted in polarity at the mastoid site. This scalp distribution, though observed on a reduced array of sensors, conforms with the classic morphology of the MMN and is identical to that observed using similar pattern paradigms [40]. The SVM_acc_ analysis demonstrated that standard tone waveforms and deviant tone waveforms could be classified with 94.5 % accuracy (as shown in Fig. 6 for the all sites feature vector at window size 900 ms). These results bode well for the use of SVMs to detect ERPs for clinical applications, such as the ERP test battery approach that has been developed by our group, known as the Halifax Consciousness Scanner (described in [19], and a companion paper, [14].

The performance of the SVM classifier was affected by a number of factors. First, the number of epochs used in the standard and deviant tone averaged waveforms was explored using a linear SVM. The accuracy of the classifier increased directly as a function of the number of epochs entered into the average. This result is entirely consistent with the general belief that the averaging process increases the SNRs of ERPs [1]. There was a consistent trend in which accuracy increased as the number of epochs entered into the average was increased from 2 to 40. The effect of number of epochs on the accuracy of the SVM was much more dramatic for serially than randomly selected epochs, and achieved a higher accuracy when using 40 epochs in the serial condition. These results suggest that sequential effects that emerge over the stimulation protocol may have contributed to the accuracy of the classifier.

The effect of averaging up to 67 epochs (the full available set) could not be explored across the group, due to the availability of less than 67 deviant epochs in some subjects as a result of artifact rejection. Nevertheless, the full set of available epochs was used for each subject in the final SVM solution, which achieved an accuracy of 94.5 %. Although the recommended number of epochs for MMN derivation is 150, with a typical recording time of 6–12 min [36], the very high accuracy that was observed here with only 67 deviants, presented in an optimized 2.5-min sequence, is promising for clinical applications. While optimal recording conditions may be difficult to achieve, particularly in a clinical environment, the relatively small amount of data required for classification using this method represents a significant reduction in testing time versus conventional stimulation parameters. Short testing times are necessary in a clinical context because they interfere minimally with routine care procedures and avoid fluctuations in vigilance, attention, and fatigue which can cause false negatives on ERP-based tests [42].

Other factors that influenced SVM accuracy were the type of kernel (linear, RBF, cubic, or quadratic), the temporal window used, and the electrode site used. When all kernel types were explored across different temporal windows, using a feature vector with data from all sites, the linear SVM clearly outperformed other kernel types. This effect was particularly noticeable as the temporal window size approached 200 ms—the period in which the MMN begins to emerge. At the maximum window size, 900 ms, the linear SVM performed significantly better than all other kernel types, particularly the RBF, which never rose above 59 %. It remains possible that optimizing the parameters of each SVM kernel type could improve performance for the nonlinear classifiers. More complex kernel types, however, can lead to overfitting, and a lack of generalizability [20]. For this reason, the effect of parameter optimization was not explored for the nonlinear classifiers.

The electrode site that was entered into the feature vector also had a significant impact on the accuracy of the classifier. In line with the known scalp distribution of the MMN [37], feature vectors from the Frontal and Central site produced significantly better results than ones from the Parietal site. Similarly, feature vectors containing data from all three scalp sites produced very high performance, which was not significantly different from the classifiers that were trained exclusively with data from the Frontal and Central sites. This approach may be preferable in clinical populations whose scalp distributions can vary from that typically observed in healthy individuals.

For the linear SVM, increasing the width of the temporal window entered into the feature vector had a direct impact on the accuracy of the classifier, in which increasing the width of the window systematically improved performance. The most dramatic improvement in accuracy occurred around 200 ms, near the peak of the MMN in the grand average (193 ms). Maximum accuracy was observed when the full post-stimulus epoch was utilized (900 ms).

One drawback of SVMs for classification, as opposed to other statistical classifiers like discriminant function analysis, is that their decision functions use complex combinations of information that can make their interpretation difficult. Thus, it is unknown which ERPs or brain oscillations that lie in the employed temporal window are contributing to the performance of the classifier. To gain insight into the waveform features that contribute most to classification accuracy, a secondary analysis (SVM_time_) was performed. Of the 46 SVMs trained in nonoverlapping 20-ms (5 sample) windows, those in the 160–260 and 720–820 ms range achieved the highest accuracy (first period maximum = 86.5 %, SD = 0.1 %, second period maximum = 83.5 %, SD = 0.058 %). The first period corresponds well with the expected latency of the MMN to the pattern deviant, which peaked on average at 193 ms. The second period corresponds with a region of positivity in the deviant tone response, which could reflect a P3-type response. While both MMN and P3 indicate that deviance detection has occurred, P3 is linked to subsequent cognitive operations like attention switching and working memory updating [43]. Regardless of the true component structure of this positive response, it is clear that a classifier which uses this portion of the waveform does not detect the MMN per se, but rather uses a larger variety of waveform features to perform the task of classifying waveforms associated with deviance detection.

In conclusion, linear SVMs proved to be effective at classifying the MMN. This method could be extremely valuable for clinical applications of the MMN, and other ERPs, by both reducing testing time versus conventional stimulation parameters and permitting the application of ERPs at the single subject level. Among the potential applications for the MMN is monitoring the status of severely brain-injured patients [15, 19, 28, 32]. Further development for use in this patient population may require additional testing to determine efficacy in the context of the severely altered EEG patterns that are commonly observed in this group [38]. Results must also be interpreted with care when applied in the absence of more concrete indicators of neurological status [44–47].
